# Editorial: *JBJS, The Bone & Joint Journal,* and *Clinical Orthopaedics and Related Research* Require Prospective Registration of Randomized Clinical Trials—Why Is This Important?

**DOI:** 10.1007/s11999-016-5174-8

**Published:** 2016-11-28

**Authors:** Seth S. Leopold, Marc Swiontkowski, Fares Haddad

**Affiliations:** 1 Clinical Orthopaedics and Related Research®, 1600 Spruce Street, Philadelphia, PA 19013 USA; 2 The Journal of Bone and Joint Surgery, Needham, MA USA; 3 The Bone & Joint Journal, London, UK

Randomized clinical trials (RCTs) represent the best study design for minimizing bias when investigating the effectiveness of a form of treatment. RCTs also are the building blocks of systematic reviews and meta-analyses, which allow more generalizable conclusions to be drawn about therapeutic efficacy [1], provided that the trials themselves are well-designed and implemented, and provided that all of the data are available for such analyses.

Unfortunately, meta-analyses can only include those data that the authors can find. Many prospective trials are started but never finished, and many more are completed but never published [2–5]. The RCTs that are published therefore only represent a proportion of those that are undertaken, and there is compelling evidence that if a study has a positive outcome it is more likely to be published [6].

Selective publication of this sort, sometimes called publication bias or positive-outcome bias, is harmful for at least two reasons. First, if two studies—one showing positive results and one showing no difference between interventions—have been performed, and the study with the positive outcome is more likely to be published and therefore available for meta-analysis, meta-analyses will tend to inflate the apparent benefits of a treatment and de-emphasize its harms [7, 8]. This may lead to the inappropriate adoption of new interventions that are less effective than they seem and that almost always are more expensive. Second, when studies that record no difference between interventions are performed but not published, a wasteful duplication of resources is likely to ensue when clinicians subsequently conduct trials that unbeknownst to them have already been carried out.

A solution to this problem has existed for some time. RCTs should be registered in one of the numerous publicly available, free, searchable, well-maintained repositories of clinical trials before the first patient is enrolled [9–11]. In addition to mitigating positive-outcome bias and reducing the likelihood that expensive, time-consuming trials will needlessly and unknowingly be repeated, prospective registration helps journals to minimize data dredging by allowing verification that the end points reported were the end points initially proposed by the researchers [12]. Prospective registration also allows patients to identify trials in which they may wish to enroll, and it can help institutional review boards find research germane to the studies that they evaluate [13].

For several years, *The Journal of Bone and Joint Surgery, The Bone & Joint Journal,* and *Clinical Orthopaedics and Related Research* ^®^ have either required or encouraged trials to be registered but permitted it to be done retrospectively (that is, after the study had been completed). We now all concur that this is not sufficient. Retrospective registration merely to allow a paper to be considered for publication by a journal does not allow the identification of no-difference findings, minimize data dredging, help patients find care, or support ethics panels in their important work. The International Committee of Medical Journal Editors (ICMJE) has required that its member journals insist on prospective registration for more than a decade now [14]. It is time for the leading journals of orthopaedic surgery to do likewise.

With that in mind, as of now, *The Journal of Bone and Joint Surgery, The Bone & Joint Journal,* and *CORR* ^®^ require registration in a publicly searchable clinical trials registry prior to the publication of RCTs, and the registry number of the trial will be published in the electronic and print versions of these papers. Until the end of 2017, which we consider a transition period, this registration may take place either prospectively or retrospectively; but as of January 1, 2018, authors of all RCTs that began after the publication of this editorial must demonstrate proof of prospective registration to be considered for review by any of our three journals.

We understand that there will be rare situations in which prospective registration is not possible or not appropriate, and we will be receptive to such claims. In these situations, the authors will be expected to explain in the limitations section of the paper the reasons for non-registration, and the editors will add a note to the title page of such a paper explaining why the exception was permitted. However, simple omission of prospective registration will not be considered a suitable excuse; the principles at stake here are too important.

We encourage all orthopaedic journals to join us in setting and maintaining this important principle underlying the reporting of research in our specialty.
